# Exclusion from the Golgi and very low levels of HTLV-2 Tax ubiquitination do not prevent IKK-gamma/NEMO relocalization and NF-κB activation

**DOI:** 10.1186/1742-4690-8-S1-A134

**Published:** 2011-06-06

**Authors:** Chloé Journo, Amandine Bonnet, Arnaud Favre-Bonvin, Jocelyn Turpin, Sébastien Chevalier, Jennifer Vinera, Emilie Côté, Claudine Pique, Renaud Mahieux

**Affiliations:** 1 Oncogenèse Rétrovirale, INSERM U758, Lyon, France; 2 Ecole Normale Supérieure, Lyon, France; 3 IFR 128 BioSciences Lyon-Gerland, Lyon, France; 4 INSERM U1016, Institut Cochin, Paris, France; 5 CNRS UMR8104, Paris, France; 6 Université Paris Descartes, Paris, France

## 

Permanent activation of the NF-κB pathway by the HTLV-1 Tax (Tax1) viral transactivator is a key event in the induction of T-cell immortalization and participates in HTLV-1-induced leukemogenesis. Tax1-induced NF-κB activation occurs through the ubiquitin-dependent recruitment of the IKK-gamma/NEMO regulatory subunit in centrosome/Golgi-associated cytoplasmic structures, which allows RelA nuclear translocation and transcription from NF-κB-dependent promoters. Although encoding a Tax protein (Tax2) that is also able to activate NF-κB, HTLV-2 does not cause leukemia. It was hence proposed that distinct Tax localizations and transactivation mechanisms could account for these differences in pathogenesis. We therefore compared the ubiquitination status of Tax2 and Tax1 as well as their ability to induce IKK-gamma/NEMO relocalization. Surprisingly, while endogenous ubiquitination of Tax1 was easily detected, endogenous Tax2 ubiquitination was barely detectable. Indeed, Tax2 ubiquitination was only seen upon ectopic expression of ubiquitin. In addition and contrary to Tax1, Tax2 was excluded from GM130-positive Golgi structures but colocalized with calreticulin, suggesting a link between Tax ubiquitination and association with the Golgi. We further showed that a non-ubiquitinable lysine-less Tax2 mutant retained the ability to induce IKK-gamma/NEMO relocalization, RelA nuclear translocation and transcription from a NF-κB-dependent promoter. Our data indicate that contrary to Tax1, Tax2 ubiquitination is dispensable for NF-κB activation. Altogether, our results reveal new significant differences between Tax1 and Tax2 and suggest an unexpected ubiquitin-independent mechanism for Tax2-induced NF-κB activation.

